# Isobaric Tags for Relative and Absolute Quantitation-Based Quantitative Serum Proteomics Analysis in Ischemic Stroke Patients With Hemorrhagic Transformation

**DOI:** 10.3389/fncel.2021.710129

**Published:** 2021-08-25

**Authors:** Zhifeng Qi, Shuhua Yuan, Xixi Zhou, Xunming Ji, Ke Jian Liu

**Affiliations:** ^1^Department of Neurology, Cerebrovascular Diseases Research Institute, Xuanwu Hospital of Capital Medical University, Beijing, China; ^2^Department of Pharmaceutical Sciences, University of New Mexico Health Sciences Center, Albuquerque, NM, United States

**Keywords:** isobaric tags for relative and absolute quantitation, serum proteomics, hemorrhagic transformation, acute ischemic stroke, biomarker

## Abstract

Hemorrhagic transformation (HT), which occurs with or without reperfusion treatments (thrombolysis and/or thrombectomy), deteriorates the outcomes of ischemic stroke patients. It is essential to find clinically reliable biomarkers that can predict HT. In this study, we screened for potential serum biomarkers from an existing blood bank and database with 243 suspected acute ischemic stroke (AIS) patients. A total of 37 patients were enrolled, who were diagnosed as AIS without receiving reperfusion treatment. They were divided into two groups based on whether they were accompanied with HT or not (five HT and 32 non-HT). Serum samples were labeled by isobaric tags for relative and absolute quantitation (iTRAQ) and analyzed by liquid chromatography coupled with tandem mass spectrometry (LC-MS/MS) and compared under NCBInr database. A total of 647 proteins in sera samples were captured, and the levels of 17 proteins (12 upregulated and five downregulated) were significantly different. These differentially expressed proteins were further categorized with Gene Ontology functional classification annotation and Kyoto Encyclopedia of Genes and Genomes metabolic pathway analysis into biological processes. Further protein–protein interaction analysis using String database discovered that, among the differentially expressed proteins, 10 pairs of proteins were found to have crosstalk connections, which may have direct (physical) and indirect (functional) interactions for the development of HT. Our findings suggest that these differentially expressed proteins could serve as potential biomarkers for predicting HT after ischemic stroke.

## Introduction

Ischemic stroke is a leading cause of death in the world. The severe complications, especially hemorrhagic transformation (HT), deteriorate the clinical condition and lead to exacerbated brain damages. HT may occur naturally or as a result of recanalization treatment (thrombolysis and/or thrombectomy). Since therapeutic interventions used during the acute phase of ischemic stroke (AIS) could significantly increase the risk of developing HT, it is essential and urgent to find biomarkers for predicting HT before recanalization treatment. Blood-based biomarker would be particularly useful, as blood can be easily obtained in emergency room. Several serum markers involving various mechanisms have been reported, such as matrix metalloproteinases (MMPs), S100 calcium-binding protein B (S-100B), cellular fibronectin (cFn), and neuron-specific enolase (NSE; Shirasaki et al., 2004; Castellanos et al., 2007; Zaheer et al., 2013; Zhong et al., 2017). However, so far, none of these serum markers is specific enough to be used in clinic to predict HT.

It was well recognized that an abnormally permeable blood–brain barrier (BBB) resulting from ischemia of the capillary endothelium allows the extravasation of blood products (Álvarez-Sabín et al., 2013). Our recent experimental study in rats showed that occludin, one of the tight junction proteins in BBB, can be cleaved into fragments after cerebral ischemia and then released into blood circulation. The level of blood occludin fragments was correlated with BBB permeability, serving as a biomarker for BBB damage (Pan et al., 2017). Our recent study in AIS patients showed that the blood occludin was significantly elevated in the early period of acute stroke (Li et al., 2020). However, the value of area under the receiver operating characteristic (ROC) curve (AUC) was less than 0.8 (0.73 in recanalization treatment and 0.77 in non-recanalization patients), suggesting that, without further improvement in occludin fragment detection specificity, blood occludin alone is not sufficient enough as a single biomarker for predicting HT. In this study, we screened for other potential serum biomarkers in serum samples from AIS patients using the isobaric tags for relative and absolute quantitation (iTRAQ)-based quantitative serum proteomics analysis.

## Materials and Methods

### Source of Clinical Data and Serum Specimens

This retrospective study was approved by the Ethics Committee of Xuanwu Hospital of Capital Medical University. We obtained the clinical data and serum specimens from our previous study (Li et al., 2020). The established database includes 243 suspected AIS patients who were administered to the emergency room at Xuanwu Hospital from November 2018 to March 2019. The Stroke Diagnosis and Treatment Quality Control and Improvement Center in Beijing, Xuanwu Hospital of Capital Medical University, is a comprehensive stroke center [acute treatments available 24/7; median door-to-needle time (DNT) < 30 min].

Blood samples were collected as soon as patients arrived at the emergency room using 21G needle and coagulated for 4 h at room temperature in Vacuette tubes (without additives, anticoagulants, and coagulant-promoting ingredients), which were routine care for stroke patients at Xuanwu Hospital. Serum was collected at 3,000 rpm for 10 min. Specimen with hemolysis was discarded. The sera were stored at −80°C without freezing and thawing for further study.

### Case Enrollment

In this study, we aim to analyze the differences of serum proteomics between HT and non-HT patients after ischemic stroke and to try to find potential biomarkers for predicting HT. HT occurrence in patients with reperfusion (thrombolysis and/or thrombectomy) could be caused by a variety of factors, including mechanical damages that vary greatly among different operators. As such, HT occurrence in patients without recanalization may better reflect the natural outcome of the disease. Therefore, for this initial study to investigate potential biomarkers, we focused on AIS patients without recanalization (*n* = 91, including five HT and 86 non-HT cases). The fundamental characteristics of enrolled 37 cases and total 91 confirmed ischemic stroke patients without reperfusion are shown in Table 1.

**TABLE 1:** Baseline characteristics of enrolled cases and cerebral infarction without reperfusion in database.

	**Cerebral infarction without reperfusion (*n* = 91)**	**Enrolled cases (*n* = 37)**	***p* value**
Age (year)	67.56 ± 13.615	64.68 ± 9.009	0.059
Male (%)	71.4%	83.8%	0.143
Median time from onset to door (h. IQR)	5.0 (2.5–11)	4.5 (2.125–10.5)	0.087
Median NIHSS score (IQR)	4 (1–10)	3 (1–6)	0.274
Median mRS score (IQR)	2 (2–3)	2 (2–3)	0.584
Atrial fibrillation (no. %)	14 (15.4%)	6 (16.2%)	0.906
Diabetes (no. %)	31 (34.1%)	7 (18.9%)	0.089
Hypertension (no. %)	57 (62.6%)	23 (62.2%)	0.960

Our previous study showed that the serum occludin concentration was a little higher in the over 80 years’ group (Li et al., 2020), suggesting that the serum components of the elderly patients may be different from those of other age patients. The five HT patients were younger than 80 years. In order to better match the HT group, we excluded the cases >80 years from the non-HT group in this study.

**FIGURE 1:**
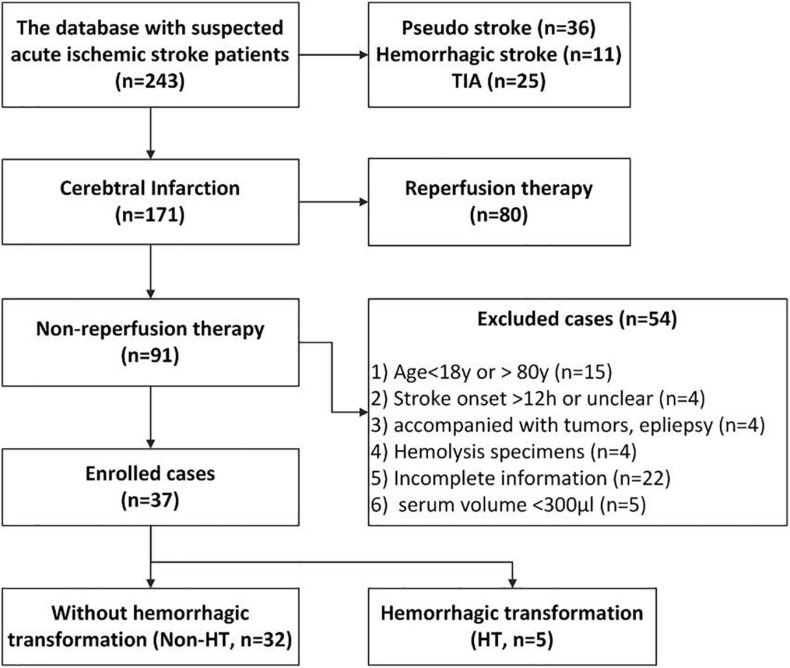
The flowchart of case enrollment. We obtained the clinical data and serum specimens from a database that was established in our previous study. This database includes 243 suspected acute ischemic stroke (AIS) patients who were administered at the emergency room at Xuanwu Hospital from November 2018 to March 2019. In this study, we enrolled the 37 AIS patients [hemorrhagic transformation (HT), *n* = 5; non-HT, *n* = 32] based on our inclusion and exclusion criteria.

A total of 37 cases (HT, *n* = 5; non-HT, *n* = 32) were finally enrolled for this study based on the inclusion and exclusion criteria below. The characteristics and related clinical information of the two groups are shown in Table 2. The flowchart of enrollment is shown in Figure 1. *Inclusion criteria*: (1) diagnosed as acute cerebral infarction by the Department of Emergency Neurology of Xuanwu Hospital and confirmed by computed tomography (CT) or MRI scan and (2) not receiving reperfusion treatments (thrombolysis and/or thrombectomy). *Exclusion criteria*: (1) age <18 or >80 years; (2) stroke onset >12 h or unclear; (3) complicating tumors and epilepsy; (4) hemolysis specimens; (5) incomplete information; and (6) available volume of serum <300 μl.

**TABLE 2:** Baseline characteristics of HT and non-HT patients in enrolled cases.

**Clinical information**	**Non-HT (*n* = 32)**	**HT (*n* = 5)**	***p* value**
Age (year)	63.81 ± 9.036	70.2 ± 7.328	0.143
Male (%)	84.4	80	0.805
Median NIHSS score (IQR)	2.5 (1–4.75)	11 (4.5–20)	0.006
Median mRS score (IQR)	2 (2–2.75)	2 (1–3.5)	0.605
Atrial fibrillation (no. %)	4 (12.5)	2 (40)	0.121
Diabetes (no. %)	6 (18)	1 (20)	0.947
Hypertension (no. %)	18 (56.3)	5 (100)	0.061
History of antiplatelets treatment (no.%)	10 (31.25%)	2 (40%)	0.698
History of anticoagulants treatment (no. %)	1 (3%)	1 (20%)	0.625
Median time from Onset to Door (h. IQR)	4 (2.125–12)	4.5 (2–11)	0.541
Median time from onset to sample (h. IQR)	4.35 (2.3–12.05)	4.6 (2.2–11.2)	0.794
Median time from onset to the first imaging (CT, h. IQR)	4.46 (2.425–12.15)	4.65 (2.275–11.275)	0.780
Median time from onset to second imaging (MRI, h, IQR)	45.2 (43.625–48.3)	43.5 (41.5–45.5)	0.176
Median time from onset to third imaging (CT, h, IQR)	143.5(135–149)	147.55(139.975–153.175)	0.237
Fibrinogen (g/L)	3.73 ± 1.075	4.53 ± 1.243	0.491
Platelet (10^9^/L)	37.02 ± 4.678	36.86 ± 4.381	0.702
Activated partial thromboplastin time (s)	204.63 ± 47.375	232.4 ± 117.594	0.628
International normalized ratio	1.05 ± 0.09	1.12 ± 0.08	0.115
Antiplatelet therapies within 48 h (no.%)			0.355
Single antiplatelet therapy	2 (6.3%)	1 (20%)	
Dual antiplatelet therapy	30 (93.8%)	4 (80%)	

### Evaluation of Hemorrhagic Transformation and National Institutes of Health Stroke Scale Score

Intracranial HT was defined as secondary intracranial bleeding following cerebral infarction, occurring naturally or related to treatment. Intracranial HT was diagnosed by the Department of Neurology of Xuanwu Hospital and confirmed by CT or MRI images since stroke onset, according to the European Cooperative Acute Stroke Study (ECASS) III criteria (Hacke et al., 2008). Briefly, stroke patients received CT scan (first imaging) to distinguish ischemic stroke and hemorrhagic stroke as soon as they arrived at the hospital. For patients with ischemic stroke, they underwent MRI examination (second imaging) for evaluation of the degree of cerebral infarction. Those who were suspected of HT would have an additional CT scan to confirm the diagnosis of HT. CT scan (third imaging) would be repeated before discharge (5–7 days). In this study, five HT cases belonged to hemorrhagic infarction-2 (HI; *n* = 4) and parenchymal hematoma-2 (PH2, *n* = 1), respectively.

National Institutes of Health stroke scale (NIHSS) score was assessed by two neurologists in the Emergency Access Department to ensure the accuracy of the assessment. Stroke severity was assessed on the basis of NIHSS score upon hospital admission and was classified as mild (0–6), moderate (7–15), and severe (≥16).

### Grouping and Serum Specimen Rearrangement

Based on HT occurrence or not within 72 h since stroke onset, the enrolled 37 cases were divided into two groups (HT, *n* = 5; non-HT, *n* = 32). Since iTRAQ method has eight different labels, it can label up to eight samples at the same time. Therefore, we matched the cases of the two groups according to the clinical information. Since the incidence of HT significantly increased in patients with severe neurological dysfunction (high NIHSS score) and long duration of ischemia (time from onset to door >6 h), the serum specimens were matched and mixed into eight samples (*n* = 4 in both groups), based on NIHSS score and stroke onset time (Seo et al., 2020). The sample rearrangement and matching are shown in Table 3.

**TABLE 3:** The sample rearrangement and labeling.

**Group**	**NIHSS score**	**Time from onset to door** **(h)**	**Number of cases (*n* = 37)**	**Sample rename for iTRAQ (*n* = 8)**	**iTRAQ** **labeling**
Non-HT	0–6	–	25	Non-HT-1	iTRAQ8-113
	7–15	<6	4	Non-HT-2	iTRAQ8-114
		>6	1	Non-HT-3	iTRAQ8-115
	≥16	–	2	Non-HT-4	iTRAQ8-116
HT	0–6	–	2	HT-1	iTRAQ8-117
	7–15	<6	1	HT-2	iTRAQ8-118
		>6	1	HT-3	iTRAQ8-119
	≥16	–	1	HT-4	iTRAQ8-121

### Abundant Protein Depletion and Quality Testing

Abundant proteins in serum, including albumin, IgG, and 10 other high-abundance proteins that can make up more than 70% of total serum protein were removed according to the instruction of Pierce Top 12 Abundant Protein Depletion Spin Columns (Thermo Fisher Scientific, Waltham, MA, United States). Samples were concentrated with 3KD ultrafiltration centrifugal tube (Millipore, Billerica, MA, United States) and replaced using urea solution (8 M, containing protease inhibitor) for three times. Total protein concentration was determined using Pierce BCA protein assay kit (Thermo Fisher Scientific). Sodium dodecyl sulfate–polyacrylamide gel electrophoresis (SDS-PAGE) gel was stained using Coomassie blue of Biofuraw^TM^ Fast Protein Stain (Tanon, Shanghai, China) to make sure that the samples meet the requirement of iTRAQ analysis (diverse proteins without degradation).

### Isobaric Tags for Relative and Absolute Quantitation Labeling and Reversed-Phase Chromatography

The sample labeling is shown in Table 3. The methods of sample digestion, iTRAQ labeling, and high pH ultra-performance liquid chromatography (UPLC) were described in previous studies (Wang et al., 2017). After reduction, alkylation, and trypsin digestion, the peptides were dried by vacuum and dissolved by 0.5 M of triethylammonium bicarbonate (TEAB). iTRAQ reagent (AB SCIEX, Redwood City, CA, United States; Lot. 4390812) was added to each tube and incubated at room temperature for 2 h. Equal amounts of labeled products were mixed in one tube and drained by vacuum concentrator. The peptides were dissolved in UPLC loading buffer (2% acetonitrile, adjusted to pH 10 with ammonia) and loaded on a reversed-phase C18 column (ACQUITY UPLC BEH C18 Column 1.7 μm, 2.1 mm × 150 mm, Waters Corporation, Milford, MA, United States) and fractionated by a ultra-high-performance LC (UHPLC) system (Thermo Scientific Vanquish Flex, Thermo Fisher Scientific, United States) at a flow rate of 200 μl/min. Then fractions were collected for LC–mass spectrometry (MS)/MS analysis.

### Liquid Chromatography Coupled With Tandem Mass Spectrometry Analysis

The fractions were then separated by EASY-nLC 1200 (Thermo Fisher Scientific, United States) and connected to Q_Exactive HF-X (Thermo Fisher Scientific, United States). Briefly, the peptides were separated by a C18 column (75 μm × 25 cm, Thermo Fisher Scientific, United States) in a linear gradient, from 0.1% formic acid/2% acetonitrile to 0.1% formic acid/80% acetonitrile at 200 nl/min for 120 min. A single Orbitrap MS scan from 350 to 1,500 *m*/*z* at a resolution of 60,000 with AGC set at 3e6 was followed by up to 20 ms/ms scans at a resolution of 15,000 with AGC set at 5e4. The top 20 were selected for fragmentation per cycle with dynamic exclusion time of 20 s.

### Protein Identification

The raw data were analyzed using ProteomeDiscoverer^TM^ Software 2.4 (Thermo Fisher Scientific, United States) and the NCBInr and SwissProt/UniProt sequence databases. A standard parameter set was used for the search, including Cys alkylation by iodoacetamide, dynamic modifications (oxidation, acetyl, iTRAQ8plex, Met-loss, Met-loss+Acetyl), *Homo sapiens*, and trypsin digestion [max. missed cleavage sites ≤2; false discovery rate (FDR) ≤ 0.01].

### Quality Control, Statistics, and Bioinformatics Analysis

In this study, blinding was applied to collect serum, clinical data, and HPLC/MS and to conduct proteomics analysis to control any potential bias. Significant difference was calculated using *t*-test: *p* < 0.05 and (fold cutoff <0.83 down-expression or >1.20 up-expression). The differential proteins were classified and analyzed using following databases: Gene Ontology (GO) for functional annotation analysis,^1^ Kyoto Encyclopedia of Genes and Genomes (KEGG) mapping for protein signal enrichment analysis,^2^ and String database^3^ for searching known and predicted protein–protein interaction network.

## Results

### Basic Clinical Information

In this initial study, to investigate potential biomarkers, we focused on AIS patients without recanalization (*n* = 91, including five HT and 86 non-HT cases). Statistical analysis showed that there was no significant difference in fundamental factors between enrolled 37 cases and total 91 confirmed ischemic stroke patients without reperfusion (Table 1). According to HT occurrence, the enrolled 37 cases were divided into two groups (HT, *n* = 5; non-HT, *n* = 32). The baseline characteristics of HT and non-HT patients in enrolled cases are shown in Table 2.

In this study, all HT occurred within 48 h and was confirmed by the second imaging scans. No new bleeding was observed by CT scan before discharge. The time from onset to the three times of imaging are shown in Table 2.

Dual antiplatelet treatments (aspirin enteric-coated tablets and clopidogrel hydrogen sulfate tablets) after the cerebrovascular event are routine therapies for confirmed cerebral infarction patients in Xuanwu Hospital (Wang et al., 2013; Zaidat et al., 2015; Johnston et al., 2018). For those who suffered from cardiogenic embolism due to atrial fibrillation, anticoagulant therapies were given at different times post-stroke onset following the management guideline of atrial fibrillation from European Heart Association and European Heart Rate Society (Kirchhof et al., 2016; Blacquiere et al., 2017; Steffel et al., 2018). In this study, there are two cases with atrial fibrillation in the HT group. They were treated with dual or single antiplatelet treatments at the beginning of hospitalization. Antiplatelet treatments were stopped as soon as signs of bleeding were discovered. In the non-HT group, there were four patients with atrial fibrillation. Three of them (NIHSS < 7) accepted anticoagulant therapy at 3 days, while the other patient (NIHSS 7–15) did not receive anticoagulant therapies during hospitalization until discharge at 7 days. The applications of antiplatelet and anticoagulant within 48 h are shown in Table 2.

Except NIHSS score, there was no significant difference in other characteristics between the two groups. For further proteomics analysis, the serum specimens were matched based on NIHSS score and stroke onset time (Table 3), making the samples comparable between the two groups.

### Screen Differentially Expressed Proteins in Serum

**FIGURE 2:**
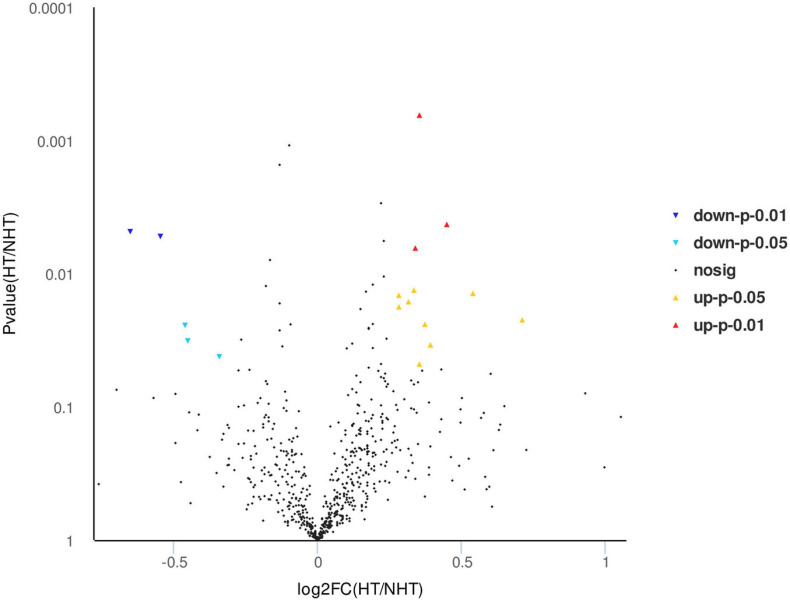
Volcano map of differential proteins between hemorrhagic transformation (HT) and non-HT groups. After isobaric tags for relative and absolute quantitation (iTRAQ) labeling and liquid chromatography coupled with tandem mass spectrometry (LC-MS/MS) identification, 17 differentially expressed proteins were quantified from a total of 647 proteins between HT and non-HT groups. The identified proteins were exported according to the log2 of fold change (HT/non-HT) and *p*-value. Yellow, upregulated under *p* < 0.05; red, upregulated under *p* < 0.01; light blue, downregulated under *p* < 0.05; blue, downregulated under *p* < 0.01; black, no significant difference proteins.

Isobaric tags for relative and absolute quantitation labeling and LC-MS/MS were used to screen differentially expressed proteins in serum between HT and non-HT samples, and a total of 647 proteins were quantified. According to the log2 of fold change (HT/non-HT) and *p*-value between the two groups, the identified proteins were exported as a volcano plot (Figure 2). It was found that 17 proteins were expressed significantly differently between the HT and non-HT groups (12 upregulated shown as red triangles and five downregulated as blue triangles), while others, which were represented as black dots, were not significantly differently expressed. The differentially expressed proteins in sera between HT and non-HT patients were shown in order of *p*-value (Table 4). The detailed proteomics data are available at the website https://www.ebi.ac.uk/pride.

**TABLE 4:** Differential expressed peptides in sera between HT and Non-HT patients.

**No.**	**Accession**	**Non-HT**	**HT**	***p* Value (HT/Non-HT)**	**Regulate**	**Description**
1	A0A4W9A917	1.05975	1.35275	0.000645596	Up	Immunoglobulin heavy constant gamma 3 (Fragment)
2	Q15848	0.92525	1.26125	0.004268125	Up	Adiponectin
3	P55083	1.21875	0.77725	0.00483628	Down	Microfibril-associated glycoprotein 4
4	Q5T0R1	0.96875	0.66375	0.005257229	Down	Adenylyl cyclase-associated protein 1 (Fragment)
5	P23142	0.98825	1.2505	0.006421342	Up	Fibulin-1
6	A0A0G2JIW1	1.12675	1.4215	0.013305522	Up	Heat shock 70 kDa protein 1B
7	Q7Z2Y8	1.13925	1.65525	0.01405189	Up	Interferon-induced very large GTPase 1
8	I3L145	0.963	1.169	0.014533687	Up	Sex hormone-binding globulin
9	S4R471	0.9655	1.20125	0.016247652	Up	Alpha-1-microglobulin (Fragment)
10	Q16270	0.97975	1.1905	0.017738997	Up	Insulin-like growth factor-binding protein 7
11	Q07075	0.76475	1.25	0.022238197	Up	Glutamyl aminopeptidase
12	P24387	1.117	1.44825	0.02400015	Up	Corticotropin-releasing factor-binding protein
13	P59665	1.07125	0.77825	0.024445556	Down	Neutrophil defensin 1
14	K7ER74	1.13975	0.83275	0.031994477	Down	Apolipoprotein C-II
15	A0A0A0MSY7	0.97975	1.28425	0.034263814	Up	Thymidine kinase 2, mitochondrial
16	Q15223	0.96525	0.76125	0.042054627	Down	Nectin-1
17	Q8TF72	0.96625	1.2325	0.04776738	Up	Protein Shroom3

### Functional Analysis of the Differentially Expressed Proteins

In order to gain global view of the functions of 17 differentially expressed proteins, the functional classification annotation and metabolic pathway analysis were performed using GO and KEGG databases, respectively.

**FIGURE 3:**
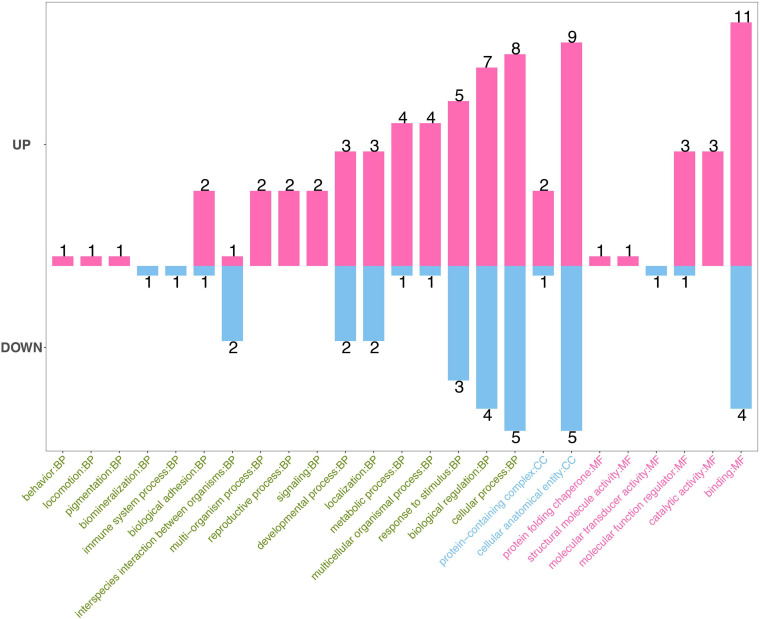
Gene Ontology (GO) enrichment analysis of differential proteins. GO enrichment analysis showed that the annotations of the differentially expressed proteins cover biological process (green), molecular function (red), and cellular component (blue). The numbers on the top of bars represent the number of annotations of the differentially expressed proteins.

Gene Ontology database can explain the functional enrichment of differential proteins and clarify the differences between samples at the functional level. GO enrichment analysis showed that the annotations of the differentially expressed proteins cover biological process, molecular function, and cellular component (Figure 3). The top five functions involve binding in molecular function (the number of proteins, 11; down, 4), cellular anatomical entity in cellular component (up, 9; down, 5), cellular process (up, 8; down, 5), biological regulation (up, 7; down, 4), and response to stimuli (up, 5; down, 3) in biological processes.

**FIGURE 4:**
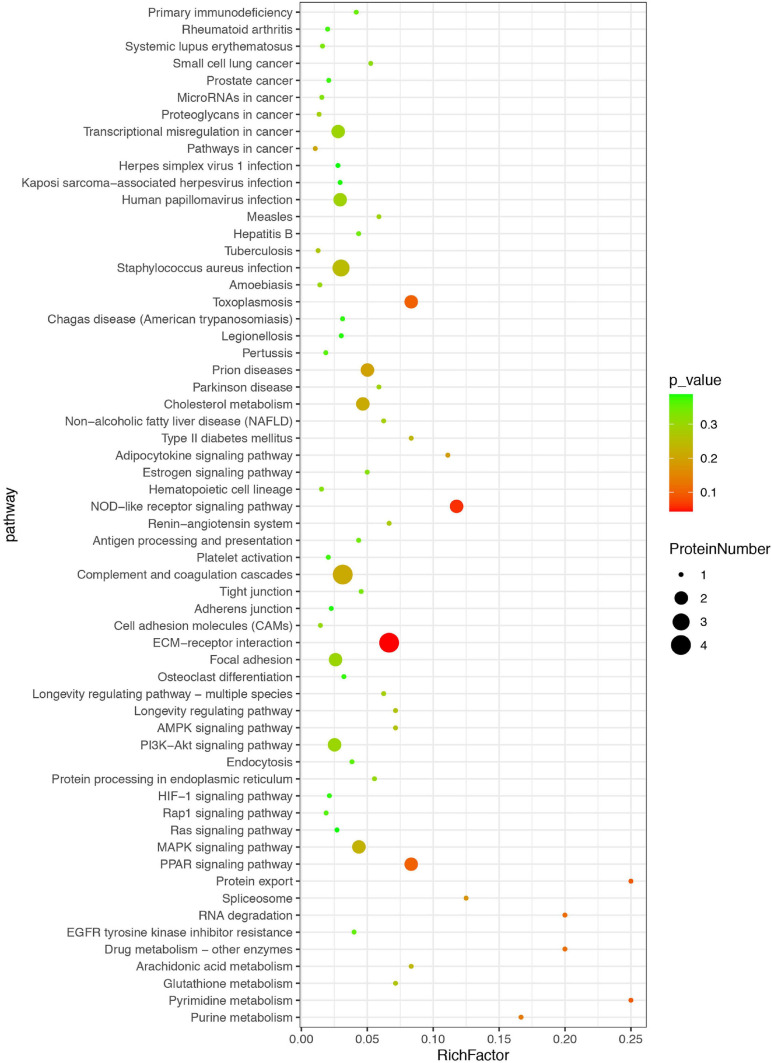
Kyoto Encyclopedia of Genes and Genomes (KEGG) enrichment analysis of differential proteins. The differentially expressed proteins were classified based on KEGG database. Each bubble represents one pathway, and the size of the bubbles reflects the protein number in each pathway. Bubble color represents the p-value of enrichment.

The differentially expressed proteins were also classified based on KEGG database, which mainly focuses on whether a group of proteins appear at a certain function. Enrichment analysis by KEGG is developed from single protein annotation analysis to protein ensemble annotation analysis in order to improve the reliability of the study and identify the most relevant biological processes of abiotic phenomena (Figure 4). The x-axis represents the ratio of enrichment (Rich factor = Sample Number/Background Number). KEGG analysis showed that the top three pathways were extracellular matrix (ECM)–receptor interaction, nucleotide binding oligomerization domain-like receptor (NOD-like receptor) pathway, and peroxisome proliferator activated-receptor (PPAR) signaling pathway, suggesting that these pathways might be more likely related to or involved in HT after ischemic stroke.

### Interaction Network Analysis of Differentially Expressed Proteins

**FIGURE 5:**
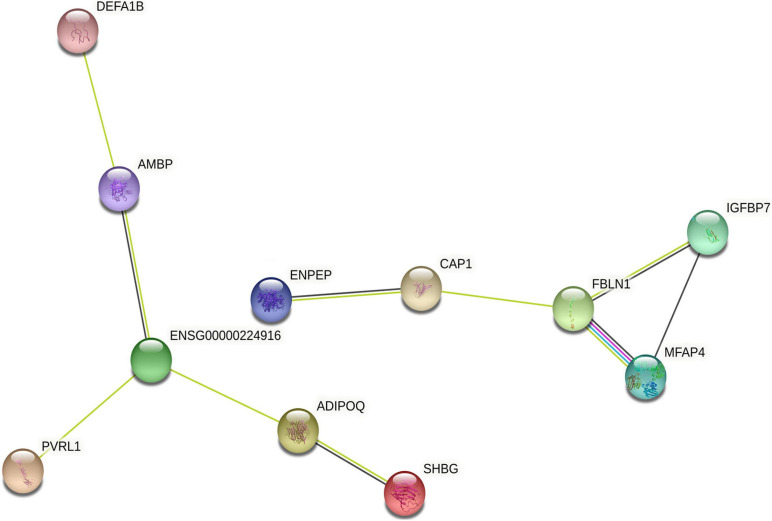
The interaction network of differentially expressed proteins. The interaction network of differentially expressed proteins was analyzed using String database. Each node represents one differential protein, and each connection reflects the interaction of proteins. The color of lines between proteins indicates the source of connection scores: co-expression (dark), experimentally determined interaction (blue), atabase annotated (pink), and automated text mining (green). The full names of the proteins are shown in Table 5.

We further analyzed the interaction network of differentially expressed proteins using String database for protein–protein interactions, including direct physical interaction and indirect functional correlation between proteins (Table 5 and Figure 5). In addition to the experimental data, the results of text mining from PubMed abstracts and other database data, and bioinformatics methods are also used to predict the interactions. A comprehensive score (0–1) was obtained according to the weights given to different methods. Among the 17 differentially expressed proteins, we found 10 pairs of proteins that have crosstalk networks. The color of lines between proteins indicates the source of connection scores: co-expression (dark), experimentally determined interaction (blue), database annotated (pink), and automated text mining (green). These data suggested that they may have direct (physical) and indirect (functional) interactions for the development of HT. It is worth noting that there are two pairs of connections with high scores (≥0.7): ADIPOQ/SHBG (Adiponectin/Sex hormone-binding globulin, comprehensive score = 0.9); FBLN1/MFAP4 (Fibulin-1/Microfibril-associated glycoprotein 4, comprehensive score = 0.7). These data suggested that the two pairs of proteins might interact with each other to play comprehensive roles in the progress of HT after ischemia stroke.

**TABLE 5:** Comprehensive score of interactions between differential expressed peptides.

**Node1**	**Node2**	**Co-expression**	**Experimentally determined interaction**	**Database annotated**	**Automated text mining**	**Total score**
ADIPOQ	ENSG00000224916	0	0	0	0.229	0.228
ADIPOQ	SHBG	0.064	0	0	0.701	0.708
AMBP	ENSG00000224916	0.214	0	0	0.151	0.304
AMBP	DEFA1B	0	0	0	0.186	0.186
CAP1	ENPEP	0.063	0	0	0.163	0.182
CAP1	FBLN1	0	0	0	0.151	0.151
ENSG00000224916	PVRL1	0	0	0	0.218	0.218
FBLN1	IGFBP7	0.098	0	0	0.178	0.227
FBLN1	MFAP4	0.23	0.064	0.9	0.311	0.943
IGFBP7	MFAP4	0.155	0	0	0	0.154

*ADIPOQ, adiponectin; AMBP, alpha-1-microglobulin/bikunin precursor; CAP1, adenylyl cyclase-associated protein 1; DEFA1B, defensin, alpha 1B; ENPEP, glutamyl aminopeptidase; ENSG00000224916, APOC4-APOC2 readthrough; FBLN1, fibulin-1; IGFBP7, insulin like growth factor-binding protein 7; MFAP4, microfibril associated glycoprotein 4; PVRL1, nectin-1; SHBG, sex hormone binding globulin.*

## Discussion

This is the first serum proteomics study focusing on differences between HT and non-HT patients after AIS. In this study, we screened a blood bank with 243 suspected AIS cases, which was established in our previous study, and enrolled 37 AIS patients (five HT and 32 non-HT). With the use of iTRAQ-based quantitative serum proteomics analysis, 17 proteins (12 upregulated and five downregulated) were found to be significantly different between HT and non-HT patients.

HT was defined as intracranial hemorrhage following cerebral infarction, which is not observed in the first but appeared in the following imaging examination. It is very important in clinic to identify patients with high risk of HT ahead of recanalization and help choose better treatments. As part of the natural process of ischemic stroke, HT can also be precipitated or enhanced by interventions in the acute phase of stroke (Yaghi et al., 2015). Recanalization therapies save penumbral tissues from deteriorating but could make the situation more complicated, such as mechanical damages in thrombectomy and activation of fibrinolysis system in thrombolysis. Studies showed that thrombolysis and/or thrombectomy significantly increased the occurrence of HT (Yaghi et al., 2017; Kim et al., 2020). Therefore, in this study, we specifically focused on the patients who did not receive recanalization treatment and identified differential serum proteins between the HT and non-HT groups. Our findings suggest that the differential serum proteins are potential biomarkers to distinguish patients with high risk of HT, who may not be suitable for recanalization treatment, or need to be more cautious about undergoing recanalization treatment. Further studies are warranted to investigate whether the potential biomarkers screened from patients without recanalization are applicable for the patients with recanalization.

In this study, we used eight-labeled iTRAQ reagents to measure 37 patient serum samples at the same time. We mixed the serum samples according to the NIHSS scores in each group, as the NIHSS score has been considered as an important risk factor for HT (Kidwell et al., 2002; Strbian et al., 2012). Adjusting the sample size of the two groups to 1:1 improves the statistical efficiency and reduces the study costs (Seo et al., 2020). However, it is worth to note that pooled mixed samples represent a strong limitation rather than an advantage in clinical studies, given the strong heterogeneity of stroke patients. Further studies need to enlarge the sample size to test the efficacy of these differential proteins for HT prediction.

Our recent animal study showed that the level of blood occludin fragments was correlated with BBB permeability after cerebral ischemia and may serve as a potential biomarker for BBB damage (Pan et al., 2017). Further pilot clinical study showed that although blood occludin significantly elevated in AIS compared with pseudo-stroke patients, blood occludin alone is not sufficient enough for HT prediction (AUC 0.73–0.77) (Li et al., 2020). In this study, the iTRAQ approach did not detect occludin fragments from the blood samples, likely due to its very low abundance in serum. On the other hand, 17 differentially expressed proteins were identified as candidates of biomarkers to predict HT. Further studies are warranted to verify the prediction accuracy of combined or comprehensive biomarkers.

In this study, KEGG metabolic pathway analysis further indicated that most of differentially expressed proteins belong to ECM–receptor interaction, NOD-like receptor pathway, and PPAR signaling pathway, involved in the progress of HT. Studies showed that ECM receptors (such as integrins and dystroglycan) NLRP3 and PPAR, which are expressed in the brain microvasculature, mediate the connections between endothelial cells and matrix components in response to cerebral ischemia/reperfusion (Tedgui and Mallat, 2001; Baeten and Akassoglou, 2011; Gong et al., 2018; Zhou et al., 2019). These studies suggest that HT may be related to vascular damages and that the proteins related to vascular endothelium may serve as biomarkers for the progression of HT. Further studies are needed to investigate the mechanism of these vascular-related proteins in HT development.

Quantitative real-time polymerase chain reaction (RT-qPCR) or enzyme linked immunosorbent assay (ELISA) is usually preformed to validate iTRAQ findings. In this study, we did not test the proteomics results using RT-PCR or ELISA. There are two main reasons: first, the differentially expressed proteins in blood may come from injured tissues (such as brain tissue). The mRNA might not correlate with the level of related proteins. Second, most of the differential proteins we identified are lacking suitable commercial antibodies or ELISA kits. Further studies with large sample size are warranted to confirm these potential biomarkers for HT and validate using self-made antibodies or ELISA kits.

This study showed that NIHSS is significantly different between the two groups (HT vs. no HT patients), which is consistent with other studies (Emberson et al., 2014; Li et al., 2020). Moreover, NIHSS score has been brought into several clinic prediction models for HT (Lou et al., 2008; Mazya et al., 2013). In order to make the samples comparable between the two groups, the serum specimens were matched based on NIHSS score and stroke onset time in this study (Table 3). On the other hand, it is less probable for a patient with a small, mild stroke to develop HT. It would be more interesting to explore in patients with moderate-to-severe stroke the differential elements between those who did or did not develop HT. Further studies are warranted to focus on the HT biomarkers especially for moderate-to-severe stroke.

Studies showed that a small amount of bleeding also affects the prognosis of patients and also needs to be analyzed (Park et al., 2012). Although asymptomatic HT did not influence the 3-month and 1-year mortality like symptomatic HT, it affected the long-term prognosis of neurological function after acute cerebral infarction, especially the long-term cognition of patients or transformed into symptomatic bleeding (Dzialowski et al., 2007; Libman et al., 2012; Lei et al., 2014). Therefore, besides symptomatic HT (PH2, according to ECASS-III criteria), we also included the patients with asymptomatic HT (HI-1, HI-2, and PH1, according to ECASS-III criteria) for analysis in this study. We will further focus on the biomarkers of symptomatic HT.

### Limitations

This is a preliminary study focusing on differences between HT and non-HT patients after AIS. There are several limitations: (1) the sample size in this retrospective study was small, and the results need to be further verified in large-size studies. (2) In order to improve the statistical efficiency and reduce the study costs, we adjusted the sample size of the two groups to 1:1 by pooled and mixed samples. The analysis of mixed sample may obscure the strong heterogeneity of stroke patients in clinic. Further studies need to enlarge the sample size to test the efficacy of these differential proteins for HT prediction. (3) In this study, we did not test the proteomics results using RT-PCR or ELISA. Further studies with large sample size are warranted to confirm these potential biomarkers for HT and to validate using self-made antibodies or ELISA kits.

In conclusion, we screened the potential serum biomarkers in AIS patients using iTRAQ-based quantitative serum proteomics analysis and identified 17 differentially expressed proteins between HT and non-HT patients. These findings suggest that these differentially expressed proteins, either alone or in combination, could be promising biomarkers for predicting HT after ischemic stroke.

## Data Availability Statement

The original contributions presented in the study are publicly available. This data can be found here: ProteomeXchange, PXD026950.

## Ethics Statement

The studies involving human participants were reviewed and approved by Ethics Committee of Xuanwu Hospital of Capital Medical University. The ethics committee waived the requirement of written informed consent for participation.

## Author Contributions

ZQ, XZ, XJ, and KL made the study design. ZQ and SY performed the experiments and data analysis. ZQ wrote the manuscript. XZ, XJ, and KL revised the manuscript. All authors contributed to the article and approved the submitted version.

## Conflict of Interest

The authors declare that the research was conducted in the absence of any commercial or financial relationships that could be construed as a potential conflict of interest.

## Publisher’s Note

All claims expressed in this article are solely those of the authors and do not necessarily represent those of their affiliated organizations, or those of the publisher, the editors and the reviewers. Any product that may be evaluated in this article, or claim that may be made by its manufacturer, is not guaranteed or endorsed by the publisher.

## Footnotes

1
http://www.geneontology.org/

2
http://www.kegg.jp/

3
http://string-db.org/
